# Men with high dark triad personality traits can accurately infer dark triad traits from other people’s faces

**DOI:** 10.3389/fpsyg.2024.1363399

**Published:** 2024-04-18

**Authors:** Keita Masui, Ryusei Yoshizumi, Hina Nakajima

**Affiliations:** Department of Psychology, Otemon Gakuin University, Osaka, Japan

**Keywords:** dark triad, face perception, Machiavellianism, psychopathy, narcissism

## Abstract

**Introduction:**

The literature suggests that people can accurately infer dark triad (DT) personality traits from other peoples’ faces. Using a self-report scale, this study investigated the impact of participants’ DT personality traits on their ability to accurately infer other peoples’ DT traits from facial cues.

**Methods:**

We created composite facial photographs of Japanese people with varying Machiavellianism, psychopathy, and narcissism scores. The Japanese participants (*N* = 170) assessed these three DT traits in the facial photographs and completed a questionnaire that assessed their own DT traits.

**Results:**

The results indicated that the participants could accurately infer all three DT traits from female faces but not from male faces. Male participants showed a positive correlation between accurate inferences of other men’s Machiavellianism and their own DT traits. In contrast, female participants showed a negative correlation between correct inferences of men’s DT traits and their own DT traits.

**Discussion:**

These findings offer novel insights into human evolutionary and social adaptations.

## Introduction

Because human beings are social animals, interacting with other people is indispensable for survival in human society. During appropriate long-term interactions with other people, it is important for individuals to be able to accurately predict personality traits and how others will think and behave. Estimating whether other parties will cooperate or exploit resources allows for the proper evaluation of potential gains and losses, which ultimately enhances people’s chances of survival. Personality psychology research has shown that human faces play a central role in social perception and cognition. They provide a rapid and rich source of information about stable social categories, such as sex, age, and race, as well as situational variables, such as physical health, emotions, and intentions, without requiring excessive cognitive effort (Parkinson, 2005; Hugenberg and Wilson, 2013). Previous studies have shown that people tend to be able to evaluate other people’s personality traits without specific sources of information, which are described as zero-acquaintance situations in the literature (Albright et al., 1988). Recent studies have suggested that people can make accurate predictions about other peoples’ personality traits solely from facial photographs, even in zero-acquaintance conditions. For example, Brown et al. (2003) showed that people can detect altruists based on nonverbal cues. Oda et al. (2009, 2021) obtained similar results using 30-s video clips of natural conversations between Japanese individuals. After watching the video clips without sound, the participants correctly estimated the target individual’s level of altruism.

Several studies have demonstrated that personality traits can be accurately estimated from facial visual information. For example, Penton-Voak et al. (2006) investigated the precision of estimating the Big Five personality traits (i.e., extraversion, neuroticism, agreeableness, conscientiousness, and openness to experience) using a neutral composite face. The participants’ neutral faces were captured, and they also completed a questionnaire to assess their own Big Five personality traits. In the first study, independent observers evaluated the levels of every facet of the Big Five personality traits by viewing participants’ facial photographs and found a significantly positive correlation between personality ratings based on photographs and self-report scores for extraversion. Moreover, the results also uncovered a positive association between personality ratings based on facial photographs and self-report scores for neuroticism and openness to experience in men. In the second study, independent observers rated the composite faces created from individuals’ self-reported high and low scores on each facet of the Big Five personality traits. The results showed that composite faces with a high degree of agreeableness and extraversion received significantly higher ratings than composite faces with low personality traits. Furthermore, Little and Perret (2007) found that extraversion and conscientiousness could be estimated accurately using composite facial photographs of both sexes. In addition, neuroticism and agreeableness were accurately estimated from the facial information of female targets. Alper et al. (2021) created high and low composite faces for each facet of the Big Five personality traits based on the self-report scale and then asked participants to make judgments about the personality traits shown in these facial photographs. The results showed that the accuracy of the inference of agreeableness and conscientiousness was significantly higher than the level of chance. In addition, the accuracy of inferring extraversion from female faces was significantly higher than chance.

Other studies have examined the relationship between facial features and the accuracy of inferring dark personality traits, such as the dark triad (DT), which includes three hostile and undesirable personality traits: Machiavellianism, psychopathy, and narcissism (Paulhus and Williams, 2002). First introduced in Niccolo Machiavelli’s book, *The Prince*, Machiavellianism is defined by the manipulation and exploitation of others, as well as cold affect, callousness, and a lack of sincerity or ethical concern (Christie and Geis, 1970). Psychopathy is characterized by hostile and aversive traits, including egocentricity, irresponsibility, shallow emotions, deficits in empathy, guilt, remorse, or morality, social deviance, manipulation of others, and high impulsivity (Hare, 2003). Narcissism is characterized by an exaggerated sense of grandiosity, superiority, entitlement, and self-worth, as well as contempt for others and an excessive need for admiration (Raskin and Terry, 1988). Although these three personality traits are conceptually distinct from each other, they do have empirically overlapping characteristics (Furnham et al., 2013). Although the DT subfactors have diverse origins, they share common malignant qualities, such as emotional coldness, self-promotion, duplicity, manipulation, and aggression (Paulhus and Williams, 2002). Jones and Figueredo (2013) argued that callousness and manipulation are common traits that have been termed the “dark core” of the three personality trait dimensions. In regard to the relationship between the DT and Big Five personality traits, all three DT traits were associated with low agreeableness (e.g., Paulhus and Williams, 2002; Jakobwitz and Egan, 2006; O’Boyle et al., 2015). In relation to the HEXACO (honesty-humility [H], emotionality [E], extraversion [X], agreeableness [A], conscientiousness [C], and openness to experience [O]) model of personality structure, all three DT traits were negatively associated with the honesty-humility factor (Lee and Ashton, 2005; Knight et al., 2018). Studies have shown that DT personality traits are associated with exploitative and socially deviant behaviors, such as aggressive behavior (Jones and Neria, 2015), internet trolling (Masui, 2019), unproductive work behaviors (O’Boyle et al., 2012), short-term mating strategies (Jonason et al., 2009), and excessive gambling (Trombly and Zeigler-Hill, 2017).

Based on self- and peer reports, Holtzman (2011) created composite faces of individuals who scored high and low on the DT personality traits. The study participants were then asked to identify the DT personality traits from these composite facial photographs, and they could accurately distinguish between individuals with high or low DT personality traits. In addition, the accuracy of inferring DT personality traits was higher for female targets than for male targets. Alper et al. (2021) replicated Holtzman’s (2011) study to determine whether DT personality traits could be identified from neutral faces using the composite facial photographs created by Holtzman (2011). Similar to Holtzman’s (2011) findings, participants could accurately distinguish individuals with high or low DT personality traits based on their facial photographs. In addition, the accuracy of inferring DT personality traits was higher than chance for both U.S. and Turkish participants. In contrast, Shiramizu et al. (2019) was only able to partially replicate these findings. They created composite facial photographs based on self-reports to measure DT personality traits and examined the accuracy of detecting DT personality traits. They revealed that inferences were accurate for the narcissism dimension in both male and female targets, whereas for the psychopathy dimension, inferences were accurate for the male targets only (Shiramizu et al., 2019). Although previous findings are not entirely consistent, examining whether people can accurately infer DT personality traits from other people’s faces may help elucidate the psychological mechanisms underlying face perception, interpersonal attraction, and the formation of appropriate interpersonal relationships. To date, how observers’ DT personality traits affect the accuracy of inferring DT personality traits from facial information has not yet been examined. Therefore, we aimed to examine this relationship.

Previous research has indicated that individuals with high DT personality traits can accurately recognize the association between facial information and associated psychological traits. For example, Del Gaizo and Falkenbach (2008) found a positive relationship between levels of psychopathy and the accuracy of recognizing negative emotions, such as fear, from facial information. Other studies have revealed an association between DT personality traits and lie-detection ability. Lyons et al. (2013) examined the relationship between psychopathy and the accuracy of detecting high-stakes emotional lies. Participants viewed a brief video clip featuring a character who was either telling the truth or lying and were subsequently asked to determine whether the character was being truthful or deceitful based on their facial information. They found a positive correlation between the accuracy of detecting lies and psychopathy among men but a negative correlation between the ability to detect lies and psychopathy among women. Furthermore, Turi et al. (2022) examined the association between DT personality traits and deception detection, with a specific focus on the role of high DT traits in deception detection because of the tendency of deceitful individuals to engage in unethical behavior. These earlier findings have suggested that individuals with higher DT personality traits can accurately detect other people’s lies, even without interacting with them. Regarding the relationship between DT personality traits and lie production, Jonason et al. (2014) found that all three DT personality traits were positively associated with lying for no reason, self-gain lies, and white lies. Taken together, current evidence suggests that those with higher DT personality traits are more accurate in identifying others’ DT personality traits from neutral facial expressions.

## Materials and methods

### Participants

This study recruited 170 Japanese participants (88 men, 82 women; mean [*M*] age = 45.8 years, range = 20–69 years, standard deviation [*SD*] = 13.9) from an internet research company (Cross Marketing, Tokyo, Japan). A medium-effect-size power analysis (*ρ* = 0.30, power of the test = 0.95) indicated that 134 participants were needed to detect significant effects.

Cross Marketing sent emails to potential candidates and invited them to participate in the study. We invited participants of varying ages to take part in this study to obtain a wide range of responses and in turn, more generalizable results. The emails explained the purpose of the survey, confidentiality and security of personal information, voluntary nature of participating in the study, freedom to withdraw from the study, and reward for participation. It also contained a link to the survey website. The potential participants were informed that the study was designed to investigate the relationship between personality traits and evaluations of the participants’ impressions of other peoples’ faces. They were given a contact address for any inquiries about the handling of personal information and were informed that if they experienced any negative effects while participating in the survey, they should close their browser immediately. They were also told that withdrawal from the study would not incur any disadvantages. The participants were required to read the study description and then click a button to access the survey. This process was considered to constitute the provision of informed consent. This survey was approved by the institutional review board of Otemon Gakuin University.

### Creating composite facial stimuli

To create the composite facial stimuli used in the face judgment task, we took photographs of 31 young adult Japanese men (*M* age = 20.5 years, *SD* = 2.0) and 28 young adult Japanese women (*M* age = 19.6 years, *SD* = 1.7). The subjects in the photographs had a neutral expression and were looking directly at the camera under normal lighting and a gray background. They then completed the Japanese version of the Dark Triad Dirty Dozen scale (DTDD-J) (Tamura et al., 2015). The distance between the camera and the targets was held constant at 2 m. Before the photo shoot, the targets removed their facial jewelry and makeup and secured their hair off their foreheads with hairpins.

The DTDD-J comprises a 12-item questionnaire that measures the DT personality traits on a 5-point Likert scale (1 = strongly disagree to 5 = strongly agree): Machiavellianism (e.g., “I tend to manipulate others to get my way”), psychopathy (e.g., “I tend to be unconcerned with the morality of my actions”), and narcissism (e.g., “I tend to want others to admire me”). The scores of the four items that assessed each DT personality trait were calculated by summing the scores of the items measuring Machiavellianism (*M* = 10.67, *SD* = 3.72), psychopathy (*M* = 10.91, *SD* = 2.97), and narcissism (*M* = 12.33, *SD* = 3.77).

**Figure 1:**
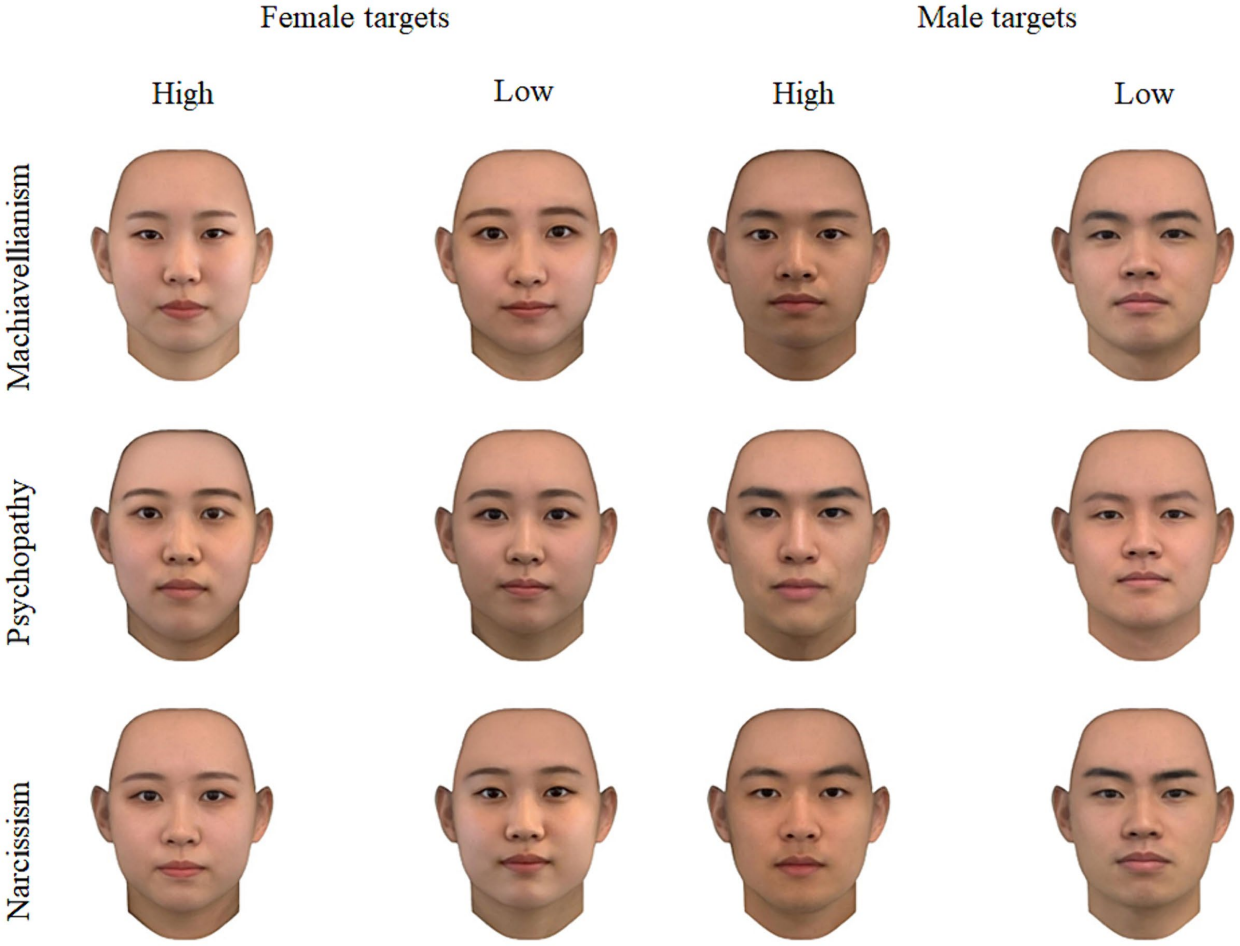
The composite facial images used in this study.

We then created prototype facial stimuli for the high and low DT personality traits by morphing five individuals who scored highest or lowest on each DTDD-J subfactor for each sex. Specifically, when we created the prototype male face with high Machiavellianism, we merged the facial photographs of five targets who scored highest on Machiavellianism. The composite face stimuli were created by averaging shape, color, brightness, and texture information for the face image using FantaMorph™ software (version 5; Abrosoft, Lincoln, NE, USA). We repeated the same process to create a total of 12 composite face stimuli, including six for male targets and six for female targets with high and low DT personality traits, respectively. Finally, we masked the hairstyles and clothing from the faces to conceal these features. Figure 1 shows the prototype faces for each DT personality trait used in the current study.

### Procedure

The participants completed the face judgment task online following the procedure in the experimental method described by Holtzman (2011). The online data collection was conducted over 2 days (October 17–18, 2023). The face judgment task comprised 12 trials, and participants were presented with two facial photographs (on the left and right of the screen) during each trial, one of which was the high DT prototype face (e.g., Machiavellianism, psychopathy, or narcissism) and the other corresponded to the low DT prototype face. The face stimuli were presented alongside a rating scale and a definition of each of the DT personality traits. For Machiavellianism, the definition was: “manipulative for personal gain, scheming, or conspiring.” For psychopathy, the definition was: “reckless, antagonistic, assertive with others, and angry at others.” For narcissism, the definition was: “arrogant, vain, pompous, self-absorbed, and assertive.” These definitions for each DT personality trait corresponded to earlier studies (Holtzman, 2011; Shiramizu et al., 2019; Alper et al., 2021). The participants completed a questionnaire using an 11-point Likert scale to indicate confidence in their ratings (−5 = confident that the person on the left matches the defined trait to +5 = confident that the person on the right matches the defined trait). The locations for the composite facial stimuli (i.e., left vs. right) were counterbalanced, and the order of trials was randomized for each participant. Following the face judgment task, the participants completed the DTDD-J to assess their levels of DT personality traits. In the current study, each DTDD-J subfactor demonstrated adequate internal consistency (Cronbach’s α > 0.63).

### Statistical analysis

To calculate the degree of the accurate inference for DT personality traits from facial photographs, we first recoded the items for which the correct response was the leftmost key on the face judgment task. For example, −5 (+5) was recoded as +5 (−5). If the correct response corresponded to the right high DT personality trait option, no recoding was necessary. This recoding procedure allowed us to interpret that a higher score on the face judgment task corresponded to higher accuracy in discriminating DT personality traits from facial photographs.

We calculated the *M*, *SD*, skewness, and kurtosis for the scores in the face judgment task and each DTDD-J subfactor. We then conducted a normality test (i.e., the Kolmogorov–Smirnov test) to examine the normality of data distribution. In addition, we compared whether there were differences in the accuracy of the ratings for each target sex and DT personality trait. Finally, the correlation coefficients between the face judgment task scores and the participants’ DTDD-J scores according to sex were calculated.

## Results

### Accuracy of inferring DT personality traits from facial photographs

We calculated the *M*, *SD*, skewness, and kurtosis for the face judgment task scores and each DTDD-J subfactor score (Table 1). The mean face judgment task score for Machiavellianism among male targets was −0.31 (*SD* = 1.54), whereas those for psychopathy and narcissism were − 0.40 (*SD* = 1.52) and − 0.33 (*SD* = 1.46), respectively. The mean face judgment task score for Machiavellianism among female targets was 0.78 (*SD* = 1.57), whereas those for psychopathy and narcissism were 0.55 (*SD* = 1.28) and 0.26 (*SD* = 1.07), respectively. The mean scores for each DTDD-J subfactor were: Machiavellianism = 2.35 (*SD* = 0.78), psychopathy = 2.75 (*SD* = 0.71), and narcissism = 2.43 (*SD* = 0.81).

**Table 1:** Means, standard deviations, skewness, and kurtosis for scores for the face judgment task and the DTDD-J.

	*M*	*SDs*	Skewness	Kurtosis
**Male targets**				
Mach.	−0.31	1.54	−0.17	1.78
Psy.	−0.40	1.52	−0.16	1.04
Nar.	−0.33	1.46	−0.35	1.56
**Female targets**				
Mach.	0.78	1.57	0.12	0.67
Psy.	0.55	1.28	0.21	2.61
Nar.	0.26	1.07	0.82	1.23
**DTDD-J**				
Mach.	2.35	0.78	−0.12	−0.87
Psy.	2.75	0.71	−0.16	0.40
Nar.	2.43	0.81	0.05	−0.55

DTDD-J, the Japanese version of the Dark Triad Dirty Dozen; Mach., Machiavellianism; Psy., psychopathy; Nar., narcissism.

The results of the Kolmogorov–Smirnov test indicated that the face judgment task scores were not distributed normally (*ps* < 0.001). A series of Kolmogorov–Smirnov tests also showed non-normal distributions for each DTDD-J subscale score (*ps* < 0.004). Thus, all subsequent analyses were conducted using nonparametric tests.

We tested whether the accuracy of inferring DT personality traits was significantly different from chance (i.e., 0) using the Wilcoxon (*W*) signed-rank sum test. For female targets, a significant difference was found for all three DT personality traits (Machiavellianism: *W* = 7,093, *p* < 0.001; psychopathy: *W* = 3,858, *p* = 0.005; narcissism: *W* = 5,747, *p* < 0.001). The accuracy of all three DT personality trait inferences for female targets was significantly above chance. For male targets, a significant difference was found for all three DT personality traits (Machiavellianism: *W* = 2,658, *p* = 0.005; psychopathy: *W* = 2,755, *p* < 0.001; narcissism: *W* = 2,767, *p* = 0.004). Therefore, the accuracy of DT personality trait inferences for male targets was significantly below chance.

Next, to explore whether the face judgment task scores differed for each sex according to the targets or DT personality traits, we conducted *W* signed-rank sum and Friedman tests. The results of the Wilcoxon signed-rank sum test revealed that the accuracy of inferring Machiavellianism (*Z* = 5.50, *p* < 0.001), psychopathy (*Z* = 5.30, *p* < 0.001), and narcissism (*Z* = 3.97, *p* < 0.001) was significantly higher for female targets than male targets. The Friedman test revealed significant differences between Machiavellianism and narcissism (*Z* = 4.20, *p* < 0.001) and psychopathy and narcissism (*Z* = 2.06, *p* = 0.04) in female targets for the accuracy of inference for each DT personality trait. The results showed that accurate inferences for DT personality traits in the female target faces were higher than those in male target faces. Furthermore, both Machiavellianism and psychopathy were more easily recognizable than narcissism in female targets.

### Relationship between the accuracy of inferring DT personality traits from faces and participants’ DT personality traits

We calculated Spearman’s rank correlation coefficients to examine the associations between the accuracy of inferring DT personality traits from faces and participants’ DT personality traits. Tables 2, 3 present the correlation coefficients between face judgment task scores and participants’ DTDD-J scores according to sex.

**Table 2:** Correlation coefficients between the accuracy of inferring DT personality traits and male participants’ DT personality traits.

	DTDD_Mach.	DTDD_Psy.	DTDD_Nar.
male_Mach.	0.37***	0.25*	0.22*
male_Psy.	−0.01	0.09	−0.02
male_Nar.	0.09	0.02	0.10
female_Mach.	−0.21*	−0.08	−0.16
female_Psy.	0.00	−0.09	0.14
female_Nar.	0.08	0.04	−0.01

DTDD, Dark Triad Dirty Dozen; Mach., Machiavellianism; Psy., psychopathy; Nar., narcissism. *p < 0.05, ***p < 0.001.

**Table 3:** Correlation coefficients between the accuracy of inferring DT personality traits and female participants’ DT personality traits.

	DTDD_Mach.	DTDD_Psy.	DTDD_Nar.
male_Mach.	−0.11	−0.28**	−0.28*
male_Psy.	−0.17	−0.14	−0.10
male_Nar.	−0.15	−0.24*	−0.24*
female_Mach.	0.07	0.08	0.11
female_Psy.	−0.06	0.03	−0.11
female_Nar.	0.14	−0.13	0.18

DTDD, Dark Triad Dirty Dozen; Mach., Machiavellianism; Psy., psychopathy; Nar., narcissism. *p < 0.05, **p < 0.01.

The results indicated that there was a positive relationship between the accurate inference for Machiavellianism in male targets and three DTDD-J subfactor scores among men (Machiavellianism: *ρ* = 0.37, *p* < 0.001, 95% confidence interval [CI] = 0.17–0.54; psychopathy: *ρ* = 0.25, *p* = 0.02, 95% CI = 0.05–0.44; narcissism: *ρ* = 0.22, *p* = 0.04, 95% CI = 0.01–0.41; Table 2). However, we observed a negative association between the accurate inference for Machiavellianism in female targets and scores for Machiavellianism (*ρ* = −0.21, *p* = 0.05, 95% CI = −0.40–0.00). For female participants, there was a negative relationship between the accurate inference for Machiavellianism in male targets and scores for psychopathy and narcissism (psychopathy: *ρ* = −0.28, *p* = 0.01, 95% CI = −0.47–0.07; narcissism: *ρ* = −0.28, *p* = 0.01, 95% CI = −0.47–0.06). In addition, there was a negative relationship between the accurate inference for narcissism in male targets and psychopathy and narcissism scores (psychopathy: *ρ* = −0.24, *p* = 0.03, 95% CI = −0.44–0.03; narcissism: *ρ* = −0.24, *p* = 0.03, 95% CI = −0.43−0.02; Table 3). The above association between participants’ DT personality traits and the accuracy of inferring DT personality traits from facial information remained significant after controlling for participant age.

## Discussion

This study aimed to explore the impact of observers’ DT personality traits on the accuracy of inferring DT personality traits from facial information. To achieve this goal, we created composite Japanese faces using photographs of individuals with high or low DT personality traits and analyzed the composite facial photographs to determine the accuracy of visually identifying DT personality traits. We examined the correlation between the accuracy of inferring DT personality traits and observers’ own DT personality traits.

### Accuracy of inferring DT personality traits from facial photographs

The face judgment task results revealed intriguing patterns in the accuracy of inferring DT personality traits from facial photographs. Notably, participants demonstrated high accuracy in identifying Machiavellianism, psychopathy, and narcissism among female targets, which is consistent with previous findings showing that DT personality traits can be deduced from facial information (Holtzman, 2011; Shiramizu et al., 2019; Alper et al., 2021). Furthermore, our results are consistent with previous research on facial cues and the Big Five personality traits, such as extraversion and conscientiousness, in female targets (Penton-Voak et al., 2006; Little and Perret, 2007; Alper et al., 2021). This suggests that female facial cues communicate information related to both favorable and unfavorable personality dimensions.

However, we found that the accuracy of inferring DT personality traits from facial photographs among men was below chance, which is inconsistent with previous findings (Holtzman, 2011; Shiramizu et al., 2019; Alper et al., 2021). This suggests that individuals found it difficult to identify DT personality traits using male facial information. To understand this unexpected result, it is important to consider the impact of individual differences in each target. In a previous study, Holtzman (2011) created a prototype face for each DT personality trait by synthesizing the faces of 10 individuals. In our study, the prototype face was synthesized from five individuals. Therefore, it is possible that in our study, the impact of each model’s facial features on the assessment of personality traits was greater than that in the previous study. As a result, the accuracy of inferring DT personality traits from facial photographs among men was below chance. It is also possible that the results of the face judgment task were influenced by a preference for physical or psychological features, such as facial features, of individuals with high DT personality traits. Previous research has shown that individuals with high DT personality traits are more likely to be rated as physically attractive by others. Holtzman and Strube (2011) hypothesized that DT personality traits evolved to exhibit higher levels of physical attractiveness. Subsequently, Holtzman and Strube (2013) confirmed this hypothesis when they found a positive association between the degree of narcissism of a photographed target and others’ ratings of the target’s physical attractiveness. In addition, Marcinkowska et al. (2015) found that women who are motivated to acquire short-term mates have a preference for highly narcissistic male faces. When men perceive the world as dangerous, they may prefer to interact with individuals who exhibit high levels of psychopathy over individuals with low levels of psychopathy (Brown et al., 2017). This suggests that psychopathy is perceived as a desirable personality trait in threatening environments. Collectively, these findings suggest that physical features, such as the face, of individuals with high DT personality traits are perceived favorably by others. This favorable perception of the physical features of individuals with high DT personality traits may explain the weaker link between socially undesirable personality traits and facial features among men observed in this study.

### Sex differences in the accuracy of inferring DT personality traits from facial photographs

Our analysis revealed sex differences in the accuracy of inferring DT personality traits from facial photographs. Machiavellianism, psychopathy, and narcissism were more accurately inferred from female targets than from male targets. These results are consistent with previous findings that the accuracy of identifying DT or Big Five personality traits from facial photographs is higher for women than men (Little and Perret, 2007; Holtzman, 2011). The observed sex differences in accuracy suggest potential variations in the expressiveness of facial features related to DT personality traits between men and women. The difference in judgments between male and female faces may be because female faces contain more cues to their actual personality than male faces. It is also possible that the sex differences in correctly identifying DT personality traits using facial information were influenced by differences in the ratings for attractiveness, masculinity, and femininity for male and female faces. Further research is needed to determine the impact of impression ratings for faces of each sex.

### Influence of observers’ DT personality traits on accuracy

When we examined the relationship between the accuracy of inferring DT personality traits and observers’ DT personality traits, we found a positive association between the accuracy of inference for Machiavellianism in male targets and male participants’ own DT personality traits. These results suggest that males who are high in each of the DT personality traits are more sensitive to others’ Machiavellian cues, which leads to more accurate inferences. In a previous study, Lyons et al. (2013) found that male individuals with high psychopathy could detect other people’s lies easily. Furthermore, Turi et al. (2022) examined the association between DT personality traits and deception detection, with a focus on the role of high DT traits in deception detection given the tendency of deceptive individuals to engage in unethical behavior. Our results showed a positive relationship between the accuracy of inferring Machiavellianism in male targets and male participants’ own DT personality traits, which expands earlier findings. In addition, individuals with high DT personality traits are characterized by deceptive and manipulative behaviors toward others to achieve their goals (Paulhus and Williams, 2002). To successfully deceive or manipulate others, individuals with high DT personality traits would need to accurately infer others’ personalities based on limited information. The positive relationship between the accuracy of inferring Machiavellianism in male targets and participants’ own DT personality traits may be a factor that contributes to the development of cunning social strategies.

In contrast, there was a negative association between the accuracy of inferring DT personality traits for male targets and female participants’ psychopathy and narcissism. These results indicate that female participants with higher levels of psychopathy and narcissism exhibited a poorer ability to accurately discern DT personality traits in male targets. On the basis of previous findings, we speculate that women with higher DT tendencies are less able to detect the lies of others (Lyons et al., 2013). However, a more detailed examination of the relationship between the accuracy of identifying DT personality traits from other people’s facial information and observers’ DT personality traits, especially in regard to sex differences, is necessary.

## Limitations and future directions

This study has several limitations. First, the race of the participants was limited to Japanese. Therefore, we cannot determine whether culture influences the ability to accurately infer DT personality traits from facial information. Some of our results are consistent with those of previous studies (Holtzman and Strube, 2011; Shiramizu et al., 2019) conducted in people of other races. For example, Alper et al. (2021) compared the U.S. and Turkish participants and found that both groups could identify DT personality traits from facial information better than chance. Future research should directly compare Japanese people and those of other races to directly examine whether the ability to accurately infer DT personality traits from facial information is influenced by culture.

Second, we were unable to clarify the effect of participants’ age on the association between facial information and the accuracy of inferring DT personality traits. Previous research has shown own-age bias in face recognition (Rhodes and Anastasi, 2012). The own-age bias refers to the tendency to discriminate faces of people of a similar age with greater accuracy than faces of younger or older people. Therefore, participant age may have impacted the accuracy of identifying DT personality traits and the association between the face judgment task and participants’ DT personality traits. However, the positive association between the face judgment task and participants’ DT personality traits was significant even after controlling for participant age. Therefore, the effect of age bias on the association between the face judgment task and participants’ DT was unlikely to be considerable. Nevertheless, it remains unclear whether age bias was present in the identification of DT traits. Further research would be needed to determine the effect of peer bias on the association between face information and the identification of DT personality traits.

Third, we did not investigate whether the specific factors that enable accurate detection of DT personality traits from facial information are innate or acquired. People may possess the ability to accurately identify others’ DT personality traits using facial information from birth, or they may develop this ability through interactions with individuals who exhibit high DT personality traits. Future research should examine the relationship between accurately identifying DT personality traits in others based on facial information and evolutionary and social adaptations.

In addition, further direct examination of the relationship between the accuracy of detecting DT personality traits from facial information and the preferences for faces of individuals with high DT personality traits is needed. Moreover, the development of interpersonal relationships with such individuals is also worth investigating. Holtzman and Strube (2013) found that individuals with high DT personality traits are perceived as more attractive during an initial encounter. However, Lyons et al. (2015) discovered that women exhibited a lower preference for facial photographs of individuals with high DT personality traits in both long- and short-term relationships. Further research should investigate how the accurate inference of DT personality traits from facial information impacts the creation and development of interpersonal relationships with individuals who have high DT personality traits.

Lastly, it is unclear which facial features are most influential in inferring DT personality traits accurately. Giacomin and Rule (2019) found that individuals can accurately identify narcissism based solely on other people’s eyes and eyebrows. Future research will be necessary to examine which specific facial features reflect different aspects of DT personalities.

In conclusion, our findings provide valuable insight into the fields of personality psychology and social cognition. The observed sex differences in inferring DT personality traits from facial photographs underscore the importance of considering contextual factors and individual differences in future research. The interplay between observers’ and targets’ personality traits raises questions about the mechanisms underlying the perception of dark personality traits or interpersonal attractiveness.

## Data availability statement

The raw data supporting the conclusions of this article will be made available by the authors, without undue reservation.

## Ethics statement

The studies involving humans were approved by the ethics committee of Otemon Gakuin University. The studies were conducted in accordance with the local legislation and institutional requirements. The participants provided their written informed consent to participate in this study. Written informed consent was obtained from the individual(s) for the publication of any potentially identifiable images or data included in this article.

## Author contributions

KM: Conceptualization, Data curation, Formal analysis, Investigation, Methodology, Writing – original draft, Writing – review & editing. RY: Conceptualization, Data curation, Investigation, Methodology, Writing – original draft, Writing – review & editing. HN: Conceptualization, Data curation, Investigation, Methodology, Writing – original draft, Writing – review & editing.
